# Immunotherapy in lung cancer.

**DOI:** 10.1038/bjc.1998.486

**Published:** 1998-08

**Authors:** M. Al-Moundhri, M. O'Brien, B. E. Souberbielle

## Abstract

More research and new treatment options are needed in all stages of lung cancer. To this end immunotherapy needs a revival in view of recent improved technologies and greater understanding of the underlying biology. In this review we discuss mechanisms of tumour immunotherapy, non-specific, specific and adoptive, with particular reference to a direct therapeutic action on all subtypes of lung cancer.


British Journal of Cancer (1998) 78(3). 282–288
© 1998 Cancer Research Campaign

Editorial

### Immunotherapy in lung cancer

**M Al-Moundhri[1], M O'Brien[1] and BE Souberbielle[1,2]**

[1]The Royal Marsden Hospital. Downs Road. Sutton. Surrey SM2 5PT: [2]Molecular Medicine. King's College School of Medicine. 123 Coldharbour Lane. London SE5 5NU. UK

**Summary** More research and new treatment options are needed in all stages of lung cancer. To this end immunotherapy needs a revival in view of recent improved technologies and greater understanding of the underlying biology.

In this review we discuss mechanisms of tumour immunotherapy, non-specific, specific and adoptive, with particular reference to a direct therapeutic action on all subtypes of lung cancer.

**Keywords:** immunotherapy; lung cancer; BCG; vaccine

### CURRENT TREATMENTS IN LUNG CANCER

Lung cancer remains the leading cause of cancer death in Western countries (Boring et al. 1993) with more than half a million new cases diagnosed annually worldwide, including 40 000 in the UK. About 80% of these tumours are of non-small-cell histological type, including squamous (40%), adeno- (40%), and large-cell carcinoma (20%). The 5-year survival of patients with non-small-cell lung cancer (NSCLC) is stage related and remains poor across all stages at about 12%. The treatment of choice for NSCLC is surgery, but only 20% of tumours are suitable for potentially curative surgery (Hoffman et al. 1980). Small-cell lung cancer accounts for the remaining 20% of lung cancer and, despite displaying initial chemosensitivity, cure is achieved in only a minority of patients.

How can survival be improved in lung cancer? Different strategies have been employed to improve the outcome. Despite the suggested benefit of adjuvant chemotherapy (NSCLC Group. 1995), the role of adjuvant therapy in operable disease awaits confirmation in large adjuvant trials. The value of preoperative (neoadjuvant) chemotherapy in NSCLC stage I. II and IIIa lung cancer is currently the focus of large randomized trials, including the MRC LU22 national study. The interest in this approach comes from the encouraging positive effect of this treatment in two randomized studies (Rosell et al. 1994; Roth et al. 1994), which have shown improved survival in patients treated with chemotherapy before surgery compared with surgery in resectable stage IIIA disease. In unresectable stage III disease there is accumulating evidence to support the use of chemotherapy before local treatment (radiotherapy or surgery), with trials showing a small survival benefit with the combined approach and improved quality of life compared with local treatment alone (Sause et al. 1995; Cullen et al. 1997). For advanced patients, chemotherapy in stage IIIb and IV disease reduces the risk of death by 27% with a survival benefit of 10% at 1 year, compared with best supportive care (NSCLC Group. 1995).

For small-cell lung cancer (SCLC) there is some optimism that more patients with limited disease will be cured with dose-intensive chemotherapy treatment (Thatcher et al. 1997). This approach is being investigated in randomized trials. However, the problem of maintaining a chemotherapy-induced remission remains and needs innovative approaches.

As in all types of lung cancer current treatment options are limited: there is thus a need to explore new treatments and with improved technology look again at older treatments such as immunotherapy. This systemic anti-tumour approach with low toxicity could form part of a panoply of future treatments in lung cancer with chemotherapy used against micrometastases, radiotherapy or surgery against local disease and possibly immunotherapy for maintenance of remissions.

### TUMOUR IMMUNOLOGY

Cancer cells differ from normal cells both qualitatively and quantitatively. These differences are due to abnormal glycosylation of surface proteins, expression of viral, mutated or overexpressed oncogene products or differentiation antigens (Boon. 1997; Weynants. 1997). Both the innate (natural killer (NK) cells, macrophages and granulocytes), and the specific arms (T and B cells) of the immune system can recognize these tumour-specific or -associated antigens (TS/AA). NK cells that detect abnormal glycosylated proteins are efficient at clearing low tumour load, especially blood-borne micrometastases, and kill cells that express a low level of HLA class I molecules. On the other hand, T cells only recognize and are stimulated by a high level of HLA molecules. They interact via their T-cell receptor with a specific peptide antigen presented on a groove of an HLA molecule. This is the first signal delivered to T cells. For T-cell activation to take place, a second signal has to be delivered via lymphokines such as interleukin (IL-2) or an interaction between the T-cell molecules (e.g. CD28) and co-stimulatory molecules (B7.1) on the antigen presenting cell (APC) (Schwartz et al. 1992). Usually, these signals are delivered via professional APC-like dendritic cells.

The fact that tumour cells are different from normal cells is not enough for efficient tumour control and, during the past few years, progress has been made in understanding the immunological escape mechanisms of tumour growth. T cells, which have the capacity of immunological memory (their response is amplified at

Received January 1998
Accepted 16 February 1998

Correspondence to: M O'Brien. The Lung Unit. Royal Marsden Hospital. Down Road. Sutton. Surrey. SM2 5PT. UK

**Table 1** Randomized adjuvant BCG in NSCLC

| Reference | Trial design | No. of patients | Comments |
|---|---|---|---|
| Jansen (1978) | Intradermal | 54 | Improved DFI in BCG group |
| Pouillart (1978) | Intradermal | 55 | Improved survival (stage I) |
| Edwards (1979) | Subdermal | 500 | No benefit |
| Miller (1979) | Oral | 308 | No benefit |
| McKneally (1981) | Intrapleural | 169 | Improved survival (stage I) |
| Mountain (1981) | Intrapleural | 473 | No benefit |
| Millar (1982) | Intradermal | 92 | No benefit |
| Ludwig Group (1986) | Intrapleural | 407 | Improved DFI in BCG group. No survival difference |

a second antigen encounter), are pivotal for any specific immune response either because they mediate the killing of the tumour cells as in the case of cytotoxic T lymphocytes (CTLs) or because they secrete cytokines, as both T helper and CTLs do, and regulate NK and CTL activation and antibody production by B lymphocytes. T-cell anergy to tumour cells could occur from the absence of tumour-specific antigens, defective antigen presentation or lack of co-stimulatory signals (Pardoll et al, 1993). Lack of tumour cell killing by CTLs could also occur if the recognition of the tumour cells by CTLs is impossible because of the lack of antigen presentation by HLA molecules. Tumour cells can probably down-regulate the expression of such molecules (Doyle et al, 1985; Korkolopoulou et al, 1996). Tumours also secrete immunosuppressive factors that may have a negative effect on T cells (Yoshino et al, 1992), e.g. SCLC cells secrete transforming growth factor beta (TGF)-β (Fischer et al, 1994) and NSCLC cells secrete a type-2 cytokine pattern (see below) (Huang et al, 1995).

Two main approaches are used to target TS/AA for tumour killing. The first is active immunotherapy, which aims to boost the anti-tumour immune response of the patient, using for example a therapeutic tumour vaccine. The second is passive immunotherapy, which bypasses the patient's immune system by administration of tumour-specific antibodies or T cells. The two approaches are not mutually exclusive and can be synergistic. In addition, a complex network of cytokines and cells regulate the immune response and any immune therapy that can influence any part of it (antigen presentation, T-cell or antibody response, cytokine production) could in theory have an effect on tumour growth. Cytokines are arbitrarily divided into type 1 [IL-2, interferon gamma (IFN-γ), IL-12], which promotes T-cell response, and type 2 (IL-4, 5, 6 and 10), which promotes antibody response (Romagnani et al, 1997). It is thought that tilting the balance towards a type 1 response is beneficial in the context of solid tumours but this rule is too simple to fit all situations. Therefore, non-specific immunomodulators that could modify the quality and the intensity of an immune response could help boost an anti-tumour effect.

### IMMUNOTHERAPY IN LUNG CANCER

### Non-specific immunostimulants

There have been several randomized clinical trials using the bacille Calmette-Guérin (BCG) vaccination in NSCLC with various administration schedules (Table 1). These trials reported mixed but mainly negative results. Although the initial trials by McKneally et al (1981) showed a statistical survival benefit for the vaccinated arm, subsequent trials failed to show any survival advantage. Similarly in SCLC, BCG vaccination following four cycles of chemotherapy showed no benefit in terms of complete response, disease-free survival or survival (Maurer et al, 1985). We are at present testing in lung cancer patients the use of *Mycobacterium vaccae* (MV), a heat-killed preparation devoid of toxicity, with a particular interest in combining this approach with chemotherapy – the rationale being that specific tumour activity may be seen after release of tumour antigens by chemotherapy combined with non-specific immunostimulation by MV (O'Brien et al, 1997).

The Ludwig Lung Cancer Group (1985) studied the administration of intrapleural *Corynebacterium parvum* in a randomized phase III trial of 475 patients with resectable lung cancer. The treated group had a significant decrease in survival. Levamisole is used in association with 5-fluorouracil (5-FU) in colon cancer but appears, overall, to make the outcome worse in lung cancer. It has been administered in different settings as shown in Table 2.

IL-2 used alone or in combination with other cytokines or lymphokine-activated killer (LAK) cells in phase II trials in NSCLC has induced some responses (Table 3). In the Eastern Co-operative Oncology group trials, IL-2 was used alone or with IFN-β: only 3 out of 73 patients showed a response, with a median survival of 35.6 weeks and no added advantage with IFN-β (Kriegel et al, 1991). Lissoni et al (1994) randomized 60 patients with advanced cancer to receive low-dose IL-2 and melatonin (pineal immunomodulating hormone) or cisplatin and etoposide chemotherapy. Although the response rates were not significantly different (24% and 19% respectively), the mean progression-free period and percentage survival were significantly different at 1 year in favour of the immunotherapy arm.

The use of IFN alone has not demonstrated activity against NSCLC, but synergy has been proposed between interferon and chemotherapy (Bowman et al, 1990). Phase II studies of interferon and chemotherapy showed response rates comparable with chemotherapy alone with acceptable toxicity (Table 4). Phase III trials using IFN alone or IFN and chemotherapy in NSCLC are shown in Table 5. These studies showed no statistically significant difference in time to progression or survival.

Randomized trials have examined the use of recombinant IFN-α as maintenance therapy following response to chemotherapy in SCLC (Table 6). All these studies showed no survival improvement for the IFN arm except for one study by Mattson et al (1992). In this study, 237 patients were randomized following chemotherapy and radiotherapy treatment to no treatment or maintenance treatment with IFN-α. A statistically significant difference in long-term survival and survival in limited stage disease was found in favour of the immunotherapy group. In conclusion, the concept of merely boosting the immune system without presentation of

© Cancer Research Campaign 1998 

Table 2 Results of Levamisole trials in treatment NSCLC

| Investigator | Study design | No. of patients | Results |
|---|---|---|---|
| Study Group for Bronchogenic Carcinoma (1975) | Operable NSCLC ± levamisole | 111 | Trend towards improved survival with levamisole |
| Amery (1978) | Levamisole administered pre- and post-operatively | 211 | Trend towards improved survival with levamisole |
| Wright (1978) | Operable NSCLC: intrapleural BCG ± levamisole | 100 | No benefit |
| Anthony (1979) | As above | 318 | significantly poorer survival |
| Pines (1980) | Inoperable squamous cell lung cancer: BCG and levamisole following RT | 50 | No benefit |
| Davis (1982) | Advanced NSCLC chemotherapy ± levamisole | 381 | No benefit |
| Holmes (1985) | Operable NSCLC: surgery ± CT or BCG and levamisole | 130 | Decreased survival with levamisole |
| Herskovic (1988) | Stage II and III. surgery + RT ± levamisole | 74 | No benefit |
| Perez (1988) | Inoperable NSCLC; radiation ± levamisole | 227 | Decreased survival with levamisole |

Table 3 IL-2 in NSCLC

| Reference | Agents used | No. of patients | Results |
|---|---|---|---|
| West (1987) | IL-2 continuous infusion | 5 | 1PR |
| Rosenberg (1989) | IL-2. IL-2/LAK or IL-2/INF | 7 | NR |
| Yang (1990) | IL-2/TNF | 16 | 1 PR |
| Jansen (1992) | IL-2/IFN-α | 11 | NR |
| Scudeletti (1993) | IL-2 intralesional and systemic | 8 | 2 PR |
| Lissoni (1993) | IL-2/melatonin | 9 | 2 PR |
| Ardizzoni (1994) | IL-2 continuous infusion | 11 | NR |

IFN-α. recombinant alpha interferon; IL-2. interleukin 2; TNF. tumour necrosis factor; LAK. lymphokine activated killer cell; PR. partial response; NR. no response

Table 4 Phase II. interferon and chemotherapy in NSCLC

| Reference | IFN/type/dose/schedule | CTX sequence | Patient no | Response/comments |
|---|---|---|---|---|
| Bowman (1990) | IFN-α 3 MU TIW or 5 MU TIW | Cisplatinum | 60 | Response rate 26% |
| Mandans (1993) | IFN-α $9 \times 10^6$ MU TIW | Carboplatin | 44 | Response rate 37% |
| Garaci (1995) | IFN-α | Cisplatin. etoposide and thymosin alpha | 56 | Overall response rate 43%. two CR |
| Kataja (1995) | IFN-α $9 \times 10^6$ units TIW | Cisplatin | 100 | Overall response rate 33% |
| Silva (1996) | IFN-α $3 \times 10^6$ unit $D_1$-$D_7$ | Cisplatin. mitomycin C. vindesine | 35 | Overall response rate 51% comparable with chemotherapy |

TIW. three times a week; IFN-α. interferon alpha; MU. million unit.

antigens is probably the reason for the overall lack of success of these approaches.

### SPECIFIC IMMUNOTHERAPY

Giving lung tumour-specific or -associated antigens (TS/AAs) has been tested using either irradiated autologous or allogenic tumour cells. tumour lysates and soluble tumour antigens. usually with an immunological adjuvant such as BCG. Studies of active specific immunization trials in lung cancer are shown in Table 7. In 1974 Hollinshead et al (1987) reported isolation of lung cancer tumour-associated antigen (TAA). A phase II study (Stewart et al. 1976) randomized patients with resectable NSCLC to receive either soluble TAA in complete Freund's adjuvant (CFA). TAA and methotrexate or no treatment post operatively. There was a significant improvement in survival (78% at 5 years) in favour of immunotherapy or chemoimmunotherapy over no treatment. Hollinshead et al (1987) reported cumulative experiences of 5-year survivals of patients entered into a phase II trial and two phase III trials of specific TAA immunotherapy. Five-year survival difference in 234 stage I and stage II NSCLC was 69% for active immunotherapy group vs 49% for control ($P = 0.0002$). Following on. a randomized trial using the same TAA was conducted. A total of 86 patients with stage I and II squamous cell carcinoma were randomized to no treatment. CFA alone or CFA + TAA with a survival of 34.5%. 53.6% and 75% respectively at 5 years. The median survival was significantly different in favour of the

 © Cancer Research Campaign 1998

**Table 5** Randomized trials of interferon in NSCLC

| Reference | Design | No. of patients | Results |
|---|---|---|---|
| Ardizzoni (1993) | Cisplatinum/epirubicin/cyclophosphamide or CEP + IFN-α | 182 | Increase response rate but no improvement in DFS or OS |
| Ciriaco (1995) | Preoperative (mitomycin, vinblastine, cisplatinum) alone or cisplatinum, etoposide, alpha thymosin and IFN-α | 110 | No significant difference in DFS or OS |
| Ardizzoni (1995) | Mitomycin C, ifosfamide, cisplatinum alone (MIP) or MIP and IFN-α cisplatinum and carboplatinum | 93 | No significant difference in DFS or OS |
| Salvati (1996) | Ifosfamide alone or ifosfamide followed by thymosin alpha and low dose IFN-α | 22 | No improvement in DFS or OS |

DFS, disease-free survival; OS, overall survival.

**Table 6** Activity of IFN as maintenance in small-cell lung cancer

| Reference | Design | No. of patients | Results |
|---|---|---|---|
| Mattson (1992) | CT + RT → CR or PR randomize to natural IFN-α or observation | 237 | Statistically significant difference in long-term survival and survival in limited group disease in favour of immunotherapy group |
| Jett (1994) | Chemotherapy + RT → randomized to observation, or IFN | 120 | Time to progression and survival inferior in patients treated with IFN |
| Tummarello (1994) | Chemotherapy → PR or CR → randomized to IFN-α or observation | 75 | No difference in response duration or survival |
| Kelly (1995) | Limited stage SCLC, following CR randomized to observation or IFN-α | 171 | No prolongation of response duration or survival |

CR, complete response; CT, chemotherapy; IFN, interferon; RT, radiotherapy; r, recombinant.

**Table 7** Randomized active vaccination trial in NSCLC

| Reference | Trial design | No. of patients | Comments |
|---|---|---|---|
| Stewart (1976) | Control, TAA, TAA and MTX | 58 | Improved DFI and overall survival |
| Perlin (1980) | No Rx, BCG alone, allogenic tumour cells + BCG | 51 | Trend in favour of immunotherapy |
| Souter (1981) | No Rx vs intradermal injection of autologous tumour cells and *C. parvum* | 80 | No survival difference |
| Stack (1982) | No Rx vs Autologous tumour cells and BCG | 83 | No survival difference |
| Hollinshead (1987) | No Rx, CFA alone, CFA + TAA | 243 | Survival advantage for immunotherapy arm (see text) |
| Price-Evans (1987) | No Rx vs irradiated autologous cells and BCG | 120 | No survival difference |
| Takita (1991) | No Rx, CFA alone TAA + CFA | 85 | Survival advantage in immunotherapy group |

TAA, tumour-associated antigen; MTX, methotrexate; CFA, complete Freund's adjuvant.

immunotherapy groups (38 months, 71 months, 106 months respectively) (Takita et al, 1991). More recently, Carbone and his colleagues (Gabrilovich et al, 1997) have vaccinated lung cancer patients with peptides encoding mutated *ras* and *p53* oncogene products. They are using the dendritic cell vaccination approach: dendritic cells are purified from cancer patients loaded with the specific peptide antigens and reinfused intravenously to the patient. The rational behind this approach is that dendritic cells are professional APCs, which express high levels of co-stimulatory molecules and HLA molecules and so an efficient T stimulation should follow after dendritic cell vaccination.

### ADOPTIVE IMMUNOTHERAPY

Rosenberg et al (1986), pioneered the use of tumour-infiltrating lymphocytes (TILs) and showed that adoptively transferred TILs exerted anti-tumour activity in patients with cancer. The ability of IL-2 to expand these cells in vitro made such an approach feasible. The initial few small trials that used adoptive immunotherapy alone or in combination with IL-2 in advanced lung cancer, demonstrated the feasibility of such an approach (Bernstein et al, 1989; Kradin et al, 1989; Faradji et al, 1991). A more recent study (Kimura et al, 1996) used adoptive immunotherapy in 82 patients

© Cancer Research Campaign 1998

following curative resection. The patients were randomized to receive IL-2 and LAKs following two courses of combination chemotherapy (cisplatin, vindesine and mitomycin) or chemotherapy alone. The 5- and 7-year survival rates of the chemo–immunotherapy group and chemotherapy group were 58.2% and 31.5% respectively in stage II and IIIA patients. This difference was statistically significant ($P = 0.0038$). In patients undergoing non-curative resection, Kimura et al (1995) reported a survival benefit for the immunotherapy arm (IL-2 and LAK) following randomization of 105 patients to chemotherapy, radiotherapy or immunotherapy. The 7-year survival rate was greater in the immunotherapy group compared with the chemotherapy and chemo–radiotherapy groups (39.1%, 12.7%, $P < 0.01$).

### FURTHER AVENUES FOR IMMUNOTHERAPY

The recent advances in tumour antigen characterization will encourage the development of more standardized anti-tumour vaccines. For example, the identification of a series of melanoma-specific gene products termed MAGEs has raised the hopes that similar specific antigens can be found in other tumours. Indeed, some of the MAGE antigens are expressed in about 40% of NSCLCs (Weynants et al, 1994)

Another approach is to provide the TS/AA via irradiated whole-cell tumour vaccines. A multitude of preclinical studies have shown that ex vivo transfection of cytokine genes [e.g. IL-2, granulocyte–macrophage colony-stimulating factor (GM-CSF)] and co-stimulatory molecule genes can augment the immunogenicity of the cell vaccine in vivo. This is improved by gene combination, e.g. *B7.1* and *IL-2* genes (Gaken et al, 1997) or *GM-CSF* and *IL-4* (Wakimoto et al, 1996).

The *IL-2* gene has been introduced into TILs via a retroviral vector to improve IL-2 delivery into the tumour. A recent phase I study used this approach in ten patients with advanced NSCLC with pleural effusion who showed some improvement in the pleural effusions (Tan et al, 1996).

Targeting the tumour by in vivo gene therapy is another option that at present is only feasible by local intratumoral delivery. It is likely that in the next 10 years progress in gene delivery systems will allow in vivo gene targeting after i.v. injection of the vector. One option is to deliver genes coding for immunostimulatory molecules such as *IL-2* (Tursz et al, 1996). Another option is to correct genetic abnormalities in tumour cells. Roth et al, (1996) have delivered a retroviral vector containing the wild-type *p53* gene directly into *p53*-mutated NSCLC tumours in nine patients with advanced disease. Wild type *p53* regulates the progression of cells in the cell cycle from $G_1$ to the S-phase. Mutation of *p53* is usually a late event in lung cancer and leads to uncontrolled growth of cancer cells. Reintroduction of the dominant wild-type (unmutated gene) can revert this process. Roth et al (1996) observed tumour regression and apoptosis in the tumours of some treated patients. Another option is to introduce a gene whose product converts a non-toxic pro-drug to a toxic compound. *Herpes simplex* virus thymidine kinase (HSV-TK) in combination with endogenous TK phosphorylates the pro-drug gancyclovir (GCV) to toxic gancyclovir triphosphate (GCV-PPP). Interestingly, GCV-PPP can enter untransfected neighbouring tumour cells through communicating gap junctions, and this leads to death of non-expressing HSV-TK cells (local bystander effect). This is important as only a small proportion (20%) of the cells in a tumour need to express HSV-TK to bring about 100% of tumour cell death. An inflammatory reaction in response to the cell death with accumulation of type-1 cytokines further increases the bystander effect by boosting local and systemic immunological recognition of the tumour cells (Freeman et al, 1997). Recently, this approach has been used in the treatment of pleural mesothelioma in rats. HSV-TK expressing adenoviral vectors were injected directly intrapleurally with significant reduction in tumour burden (Elshami et al, 1996). Human studies are on-going (Treat et al, 1996).

Another approach is to use anti-idiotypic antibodies. These antibodies are raised against monoclonal antibodies recognizing cell-surface tumour antigen and have a similar shape to the tumour antigen. This approach is currently the focus of an EORTC trial (SILVA study) that uses an anti-idiotype BEC2 (anti-idiotype to ganglioside GD3) combined with BCG adjuvant in SCLC. A pilot study (Grant et al, 1996) using BEC2/BCG in patients with SCLC showed minimal toxicity, with median survival not reached after 15 months, which compares favourably with historic controls.

### CONCLUSION

Overall outcome from standard treatments for lung cancer remains poor. Immunotherapy could have an important role to play in the treatment of lung cancer. Active specific vaccination is safe to administer and available data suggest beneficial effect in the adjuvant setting; recent advances in tumour antigen characterization and gene therapy will aid the design of more effective vaccines.

### REFERENCES

Amery WK (1978) Final results of a multicenter placebo-controlled Levamisole study of resectable lung cancer. *Cancer Treat Rep* **62(11)**: 1677–1683

Anthony HM, Mearns AJ, Mason MK, Scott DG, Moghissi K, Deverall PB, Rozycki ZJ and Watson DA (1979) Levamisole and surgery in bronchial carcinoma patients: increase in deaths from cardiorespiratory failure. *Thorax* **34**: 4–12

Ardizzoni A, Salvati F, Rosso R, Bruzzi P, Rubagotti A, Pennucci MC, Mariani GL, De Marinis F, Pallotta G and Antilli A (1993) Combination of chemotherapy and recombinant alpha-interferon in advanced non small cell lung cancer. Multicentric Randomized FONICAP Trial Report. The Italian Lung Cancer Task Force. Cancer **72**: 2929–2935

Ardizzoni A, Bonavia M, Vialke M, Baldini E, Mereu C, Verna A, Ferrini S, Cinquegrana A, Molinari S and Mariani GL (1994) Biologic and clinical effects of continuous infusion interleukin-2 in patients with non small cell lung cancer. *Cancer* **73**: 1353–1360

Ardizzoni A, Addamo GF, Baldini E, Borghini U, Portalone L, De-Marinis F, Lionetto R, Conte PF, Bruzzi P and Pennucci MC (1995) Mitomycin–ifosfamide–cisplatinum (MIP) vs MIP–interferon vs cisplatinum–carboplatin in metastatic non small cell lung cancer: a FONICAP randomised phase II study. Italian Lung Cancer Task Force. *Br J Cancer* **71**: 115–119

Bernstein Z, Goldrosen M and Krajewski C (1989) Phase II trial of IL-2/LAK therapy for patients with HD/NHL and NSC lung cancer (abstract). *Proc Am Soc Clin Oncol* **8** A754

Boon T, Coulie PG and Van den Eynde B (1997) Tumour antigens recognized by T cells. *Immunol Today* **18**: 267–268

Boring CC, Squires TS and Tong T (1993) Cancer Statistics 1993. *Ca Cancer J Clin* **43**: 7–26

Bowman A, Fergusson RJ, Allan SG, Stewart ME, Gregor A, Cornbleet MA, Greening AP, Crompton GK, Leonard RC and Smyth JF (1990) Potentiation of cisplatin by alpha-interferon in advanced non small cell lung cancer (NSCLC): a phase II study. *Ann Oncol* **1**: 351–353

Ciriaco P, Rendina EA, Venuta F, De Giacomo T, Della-Rocca G, Flaishman I, Baroni C, Cortesi E, Bonsignore G and Ricci C (1995) Preoperative chemotherapy and immunochemotherapy for locally advanced stage IIIA and IIIB non small cell lung cancer. Preliminary results. *Eur J Cardiothoracic Surg* **9**: 305–309

Cullen MH, Billingham LJ, Woodroffe CM, Billingham LJ, Chetiyawardana AD, Joski R, Ferry D, Connolly CK and Bessell E (1997) Mitomycin, ifosphamide and cisplatin (MIC) in non-small cell lung cancer (NSCLC): results of a

© Cancer Research Campaign 1998

randomised trial in patients with localised, inoperable disease (abstract). *Lung* **18** (Suppl. 1): 5

Davis S. Mietlowski W. Rohwedder JJ. Griffin JP and Neshat AA (1982) Levamisole as an adjuvant to chemotherapy in extensive bronchogenic carcinoma: a Veterans Administration Lung Cancer Group Study. *Cancer* **50**: 646–651

Doyle A. Martin WJ. Funa K. Gazdar A. Carney D. Martin SE. Linnolia I. Cuttitta F. Malshire J and Bunn P (1985) Markedly decreased expression of class I histocompatibility antigens, protein, and mRNA in human small-cell lung cancer. *J Exp Med* **161**: 1135–1151

Edwards FR (1979) Use of BCG as an immunostimulant after resection of carcinoma of the lung: a two-year assessment of a trial of 500 patients. *Thorax* **34**: 801–806

Elshami AA. Kucharczuk JC. Zhang HB. Smythe WR. Hwang HC. Litzky LA. Kaiser LR and Albelda SM (1996) Treatment of pleural mesothelioma in an immunocompetent rat model utilizing adenoviral transfer of the herpes simplex virus thymidine kinase gene. *Hum Gene Therapy* **7**: 141–148

Faradji A. Bohbot A. Schmitt-Goguel M. Roselin N. Dumont S. Wiesel ML. Lallot C. Eber M. Bartholeyns J and Poindron P (1991) Phase I trial of intravenous infusion of ex vivo-activated autologous blood derived macrophages in patients with non small cell lung cancer: toxicity and immunomodulatory effects. *Cancer Immunol Immunother* **33**: 319–326

Fischer JR. Darjes H. Lahm H. Schindel M. Drings P and Krammer PM (1994) Constitutive secretion of bioactive transforming growth factor b1 by small cell lung cancer cell lines. *Eur J Cancer* **30**: 2125–2129

Freeman SM. Ramesh R and Marrogi AJ (1997) The 'bystander effect': tumour regression when a fraction of the tumour mass is genetically modified. *Lancet* **349**: 2–3

Gabrilovich D. Kavanaugh D. Ishida T. Oyama T. Taek C. Nadaf S. Sepetavec T. Jensen R. Gazdar A. Ciernik F. Corak J. Berzofsky J and Carbone DP (1997) Induction of p53/ras specific cellular immunity in patients with common solid tumors. *Lung* **18** (Suppl. 2): 90–91

Gaken JA. Hollingsworth SJ. Hirst WJ. Buggins AG. Galea-Leuri J. Peakman M. Kuiper M. Patel P. Towner P. Patel PM. Collins MK. Mufti GJ. Farzaneh FF and Darling DC (1997) Irradiated NC adenocarcinoma cells transduced with both B7.1 and interleukin-2 induce CD4+ mediated rejection of established tumours. *Human Gene Therapy* **8**: 477–488

Garaci E. Lopez M. Bonsignore G. Della-Giulia M. D'Aprile M. Favalli C. Rasi G. Santini S. Capomolla E and Vici P (1995) Sequential chemoimmunotherapy for advanced non small cell lung cancer using cisplatin, etoposide, thymosin-alpha 1 and interferon-alpha 2a. *Eur J Cancer* **31A**. 2403–2405

Grant SC. Yao TJ. Kris MG. Rigas JR. Pisteris KMW. Miller V. Houghton AN and Chapman PB (1996) Long survival following immunisation with BEC2 plus BCG after initial therapy for small cell lung cancer (SCLC). *Proceedings of American Society of Clinical Oncology*. **155**: 555 (A1806)

Hahne M. Rimoldi D. Schroter M. Romero P. Schreier M. French LE. Schneider P. Bornard T. Fontana A. Lienard D. Cerottini J and Tschopp J (1996) Melanoma cell expression of Fas (Apo-1/CD95) ligand: implications for tumour immune escape. *Science* **274**: 1363–1366

Hoffman TM and Randsell HT (1980) Comparison of lobectomy and wedge resection for carcinoma of lung. *J Thorac Cardiovasc Surg* **79**: 211–217

Herskovic A. Bauer M. Seydel HG. Yesner R. Doggett RL. Perez CA. Durbin LM and Zinninger M (1988) Post-operative thoracic irradiation with or without Levamisole in non-small cell lung cancer: results of a Radiation Therapy Oncology Group Study. *Int J Radiat Oncol Biol Phys* **14**: 37–42

Hollinshead A. Stewart TH. Takita H. Dalbow M and Concannon J (1987) Adjuvant specific active lung cancer immunotherapy trials. Tumor-associated antigens. *Cancer* **60**: 1249–1262

Holmes EC. Hill LD and Gail M (1985) A randomized comparison of the effects of adjuvant therapy on resected stages II and III non-small cell carcinoma of the lung. The Lung Cancer Study Group. *Ann Surg* **202**(3): 335–341

Huang M. Wang J. Lee P. Sharman S. Mao JT. Meissner H. Uyemura K. Modlin R. Wollman J and Dubinett SM (1995) Human non-small cell lung cancer cells express a type 2 cytokine pattern. *Cancer Res* **55**: 3847–3853

Jansen HM. The TM. de Gast GC. Esselink MT. vanderwal AM and Orie NG (1978) Adjuvant immunotherapy with BCG in squamous cell bronchial carcinoma: immune reactivity in relation to immunostimulation. (Preliminary results in a controlled trial). *Thorax* **33**: 429–438

Jansen RL. Slingerland R. Goey SH. Franks CR. Bolhuis RL and Stoter G (1992) Interleukin-2 and interferon-alpha in the treatment of patients with advanced non small cell lung cancer. *J Immunother*. **12**: 70–73

Jett JR. Maksymiuk AW. Su JQ. Mailliard JA. Krook JE. Tschetter LK. Kardinal CG. Twito DI. Levitt R and Gerstner JB (1994) Phase III trial of recombinant interferon gamma in complete responders with small cell lung cancer. *J Clin Oncol* **12**: 2321–2326

Kataja V and Yap A (1995) Combination of cisplatin and interferon-alpha 2a (Roferon-A) in patients with non small cell lung cancer (NSCLC). An open phase II multicentre study. *Eur J Cancer* **31A**: 35–40

Kelly K. Crowley JJ. Bunn PA Jr. Hazuka MB. Beasley K. Upchurch C. Weiss GR. Hicks WJ. Gandara DR and Rivkin S (1995) Role of recombinant interferon alfa-2a maintenance in patients with limited stage small cell lung cancer responding to concurrent chemoradiation: a Southwest Oncology Group Study. *J Clin Oncol* **13**: 2924–2930

Kimura H and Yamaguchi Y (1995) Adjuvant immunotherapy with interleukin-2 and lymphokine-activated killer cells after noncurative resection of primary lung cancer. *Lung Cancer* **13**(1): 31–44

Kimura H and Yamaguchi Y (1996) Adjuvant chemo-immunotherapy after curative resection of Stage II and IIIA primary lung cancer. *Lung Cancer* **14** (2–3): 301–314

Korkolopoulou P. Kaklamanis L. Pezella L. Harris AL and Gatter KC (1996) Loss of antigen-presenting molecules (MHC class I and TAP-1) in lung cancer. *Br J Cancer* **73**: 148–153

Kradin RL. Kurnick JT. Lazarus DS. Preffer FL. Dubinett SM. Pinto CE. Gifford J. Davidson E. Grove B and Callahan RJ (1989) Tumour-infiltrating lymphocytes and interleukin-2 in treatment of advanced cancer. *Lancet* **1**: 577–580

Kriegel R. Lynch E. Kucuk O. Tester W. Chang A. Bonomi P. Huberman M. Padavic K. Dutcher J. Bium R and Comis R (1991) Interleukin-2 (IL-2) therapies prolong survival in metastatic non small cell lung cancer (NSCLC) (abstract 844). *Proc Am Soc Clin Oncol* **10**: 246

Lissoni P. Barni S. Rovelli F. Brivio F. Ardizzoia A. Tancini G. Conti A and Maestroni GJ (1993) Neuroimmunotherapy of advanced solid neoplasms with single evening subcutaneous injection of low dose interleukin-2 and melatonin: preliminary results. *Eur J Cancer* **29A**: 185–189

Lissoni P. Meregalli S. Fossati V. Paolorossi F. Barni S. Tancini G and Grigerio F (1994) A randomised study of immunotherapy with low dose subcutaneous interleukin-2 plus melatonin vs chemotherapy with cisplatin and etoposide as first line therapy for advanced non small cell lung cancer. *Tumori* **8**: 464–467

Ludwig Lung Cancer Study Group (1985) Adverse effect of intrapleural *Corynebacterium parvum* as adjuvant therapy in resected stage I and II non small cell carcinoma of the lung. *J Thorac Cardiovasc Surg* **89**: 842–847

Ludwig Cancer Study Group (LLCSG) (1986) Immunostimulation with intrapleural BCG as adjuvant therapy in resected non-small cell lung cancer. *Cancer* **58**: 2411–2416

McKneally MF. Maver C. Lininger L. Kausel HW. McIlduff JB. Older TM. Foster ED and Alley RD (1981) Four-year follow-up on the Albany experience with intrapleural BCG in lung cancer. *J Thorac Cardiovasc Surg* **81**: 485–492

Mandanas R. Einhorn LH. Wheeler B. Ansari R. Lutz T and Miller ME (1993) Carboplatin (CBDCA) plus alpha interferon in metastatic non small cell lung cancer. A Hoosier Oncology Group phase II trial. *Am J Clin Oncol* **16**: 519–521

Mattson K. Niiranen A. Pyrhonen S. Holsti LR. Holsti P. Kumpulainen E and Cantell K (1992) Natural interferon alfa as maintenance therapy for small cell lung cancer. *Eur J Cancer* **28A**: 1387–1391

Maurer LH. Pajak T. Eaton W. Comis R. Chahinian P. Faulkner C. Silberfarb PM. Henderson E. Rege VB and Baldwin PE (1985) Combined modality therapy with radiotherapy, chemotherapy and immunotherapy in limited small cell carcinoma of the lung: a Phase III cancer and Leukemia Group B Study. *J Clin Oncol* **3**: 969–976

Millar JW. Roscoe P. Pearce SJ. Ludgate S and Horne NW (1982) Five-year results of a controlled study of BCG immunotherapy after surgical resection in bronchogenic carcinoma. *Thorax* **37** (1): 57–60

Miller AB. Taylor HE. Baker MA. Dodds DJ. Falk R. Frappier A. Hill DP. Jindani A. Landi S. Macdonald AS. Thomas JW and Wall C (1979) Oral administration of BCG as an adjuvant to surgical treatment of carcinoma of the bronchus. *Can Med Assoc J* **121** (1): 45–54

Mountain CF and Gail MH (1981) Surgical adjuvant intrapleural BCG treatment for stage I non small cell lung cancer. *J Thorac Cardiovasc Surg* **82**: 649–657

Non-small Cell Lung Cancer Collaborative Group (1995) Chemotherapy in non-small cell lung cancer: a meta-analysis using updated data on individual patients from 52 randomised clinical trials. *Br Med J* **311**: 899–909

O'Brien MER. Bromelow K. Prendiville J. Rees C. Hill M. Stanford J. Grange J. Farzaneh F. Smith I E and Souberbielle BE (1997) A study of SRL 172 (*Mycobacterium vaccae*) as the first component of a tumour vaccine with immunological changes and clinical activity in patients with lung cancer (abstract). *Lung* **18** (suppl. 1): 163

Pardoll DM (1993) Cancer vaccines. *Immunol Today* **14**: 310–316

Perez CA. Bauser M. Emami BN. Byhardt R. Brady LW. Doggett RL. Gardner P and Zinninger M (1988) Thoracic irradiation with or without Levamisole (NSC # 177023) in unresectable non-small cell carcinoma of the lung: a phase III randomized trial of the RTOG. *Int J Radiat Oncol Biol Phys* **15**: 1337–1346

Perlin E. Oldham RK. Weese JL. Heim W. Reid J. Mills M. Miller C. Blom J. Green D. Bellinger S Jr. Cannon GB. Law I. Connor R and Herberman RB (1980) Carcinoma of the lung: immunotherapy with intradermal BCG and allogeneic tumour cells. *Int J Radiat Oncol Biol Phys* **6**: 1033–1039

Pines A (1980) BCG plus Levamisole following irradiation of advanced squamous bronchial carcinoma. *Int J Radiat Oncol Biol Phys* **6**: 1041–1042

Pouillart P. Mathe G. Palangie T. Schwarzenberg L. Huguenin P. Morin P. Gautier H and Baron A (1978) Trial of BCG immunotherapy in the treatment of resectable squamous cell carcinoma of the bronchus (stages I and II). *Recent Results Cancer Res* **62**: 151–155

Price-Evans DA. Roberts HL. Hewitt S. Walsh D. Donohue WTA and Lambourne A (1987) A trial of adjuvant immunotherapy for bronchial carcinoma with irradiated autologous tumour cells. *Int J Immunother* **3**: 293

Romagnani S (1997) The Th1/Th2 paradigm. *Immunol Today* **18**: 263–266

Rosell R. Gomez Codina J. Camps C. Maestre J. Padille J. Canto A. Mate JL. Li S. Roig J and Olazabal A (1994) A randomized trial comparing preoperative chemotherapy plus surgery with surgery alone in patients with non-small-cell lung cancer. *N Engl J Med* **330**: 153–158

Rosenberg SA. Spiess P and Lafreniere R (1986) A new approach to the adoptive immunotherapy of cancer with tumor-infiltrating lymphocytes. *Science* 233 **4770**: 1318–1321

Rosenberg SA. Lotze MT. Yang JC. Aebersold PM. Linehan WM. Seipp CA and White DE (1989) Experience with the use of high dose interleukin-2 in the treatment of 652 cancer patients. *Ann Surg* **210**: 474–484 (discussion 484–485)

Roth JA. Fossella F. Komaki R. Ryan MB. Putnam JB Jr. Lee JS. Dhingra H. De Caro L. Chasen M and McGravran M (1994) A randomized trial comparing perioperative chemotherapy and surgery with surgery alone in resectable stage IIIA non-small-cell lung cancer. *J Natl Cancer Inst* **86**: 673–680

Roth JA. Nguyen D. Lawrence DD. Kemp BL. Carrasco CH. Ferson DZ. Hong WK. Komaki R. Lee JJ. Nesbitt JC. Pisters KM. Putnam JB. Schea R. Shin DM. Walsh GL. Dolormente MM. Han CI. Martin FD. Yen N. Xu K. Stephens LC. McDonnell TJ. Mukhopadhyay T and Cai D (1996) Retrovirus-mediated wild-type p53 gene transfer to tumors of patients with lung cancer. *Nat Med* **2(9)**: 985–991

Salvati F. Rasi G. Portalone L. Antilli A and Garaci E (1996) Combined treatment with thymosin-alpha 1 and low dose interferon-alpha after ifosfamide in non small cell lung cancer: a phase II controlled trial. *Anticancer Res* **16**: 1001–1004

Sause WT. Scott C. Taylor S. Johnson D. Livingston R. Komaki R. Emami B. Curran WJ. Byhardt RW and Turrisi AT (1995) Radiation Therapy Oncology Group (RTOG) 88–08 and Eastern Cooperative Oncology Group (ECOG) 4588: preliminary results of a phase III trial in regionally advanced, unresectable non small cell lung cancer. *J Natl Cancer Inst* **87**: 198–205

Schwartz RH (1992) Costimulation of T lymphocytes: the role of CD28, CTLA-4, and B7/BB1 in interleukin-2 production and immunotherapy. *Cell* **71**: 1065–1068

Scudeletti M. Filaci G. Imro MA. Motta G. Di-Gaetano M. Perri I. Tongiani S. Indiveri F and Puppo F (1993) Immunotherapy with intralesional and systemic interleukin-2 of patients with non small cell lung cancer. *Cancer Immunol Immunother* **37**: 119–124

Silva RR. Bascioni R. Rossini S. Zuccatosta L. Mattioli R. Pilone A. Delprete S. Battelli N. Gasparini S and Battelli T (1996) A phase II study of mitomycin C, vindesine and cisplatin combined with alpha interferon in advanced non small cell lung cancer. *Tumori* **82**: 68–71

Souter RG. Gill PG. Gunning AJ and Morris PJ (1981) Failure of specific active immunotherapy in lung cancer. *Br J Cancer* **44**: 496

Stewart TH. Hollinshead AC. Harris JE. Belanger R. Crepeau A. Hooper GD. Sachs HJ. Klaassen DJ. Hirte W. Rapp E. Crook AF. Orizaga M. Sengar DP and Raman S (1976) Immunochemotherapy of lung cancer. *Ann NY Acad Sci* **277**: 436–466

Study Group for Bronchogenic Carcinoma (1975) Immunopotentiation with Levamisole in resectable bronchogenic carcinoma: a double-blind controlled trial. *Br Med J* **3** (5981): 461–464

Stack BHR. McSwan N and Stirling JM (1982) Autologous x-irradiated tumour cells and percutaneous BCG in operable lung cancer. *Thorax* **37**: 588

Takita H. Hollinshead AC. Adler RH. Bhayana J and Ramundo M (1991) Adjuvant, specific, active immunotherapy for resectable squamous cell lung carcinoma: a 5-year survival analysis. *J Surg Oncol* **46**: 9–14

Tan Y. Xu M. Wang W. Zhang F. Li D. Xu X. Gu J and Hoffman RM (1996) IL-2 gene therapy of advanced lung cancer patients. *Anticancer Res* **16** (4A): 1993–1998

Thatcher N. Sambrook RJ and Stephens RJ The MRC Lung Cancer Working Party (1997). First results of a randomised trial of dose intensification with G-CSF in small cell lung cancer (abstract). *Lung* 18 (**Suppl. 1**): 7

Treat J. Kaiser LR and Storman DH (1996). Treatment of advanced mesothelioma with the recombitant adenovirus H. H5. 010RSVTK. A phase I trial (BB-IND 6274). *Human Gene Therapy* **7**: 2047–2057

Tummarello D. Graziano F. Mari D. Cetto G. Pasini F. Antonio S. Isidori P and Gasparini S (1994) Small cell lung cancer (SCLC): a randomised trial of cyclophosphamide, adriamycin, vincristine plus etoposide (CAV-E) or teniposide (CAV-T) as induction treatment, followed in complete responders by alpha-interferon or no treatment, as maintenance therapy. *Anticancer Res* **14**: 2221–2227

Tursz T. Cesne AL. Baldeyrou P. Gautier E. Opolon P. Schatz C. Pavirani A. Courtney M. Lamy D. Ragot T. Saulnier P. Andremont A. Monier R. Perricaudet M and Le Chevalier T (1996) Phase I study of a recombinant adenovirus mediated gene transfer in lung cancer patients. *J Natl Cancer Inst* **88**: 1857–1863

Wakimoto H. Abe J. Tsunoda R. Aoyagi M. Hirakawa K and Hamada H (1996) Intensified antitumour immunity by a cancer vaccine that produces granulocyte-macrophage colony-stimulating factor plus interleukin 4. *Cancer Res* **56**: 1828–1833

West WH. Tauer KW. Yannelli JR. Marshall GD. Orr DW. Thurman GB and Oldham RK (1987) Constant-infusion recombinant interleukin-2 in adoptive immunotherapy of advanced cancer. *N Engl J Med* **316**: 898–905

Weynants P. Lethe B. Brasseur F. Marchand M and Boon T (1994) Expression of MAGE genes by non-small-cell-lung carcinomas. *Int J Cancer* **56**: 826–829

Weynants P. Marchandine FX and Sibille Y (1997) Pulmonary perspective: immunology in diagnosis and treatment of lung cancer. *Eur Respir J* **10**: 1703–1719

Wright PW. Hill Liposomal Doxorubicin. Peterson AV Jr. Pinkham R. Johnson L. Ivey T. Bernstein I. Bagley C and Anderson R (1978) Preliminary results of combined surgery and adjuvant Bacillus Calmette-Guerin plus Levamisole treatment of resectable lung cancer. *Cancer Treat Rep* **62**: 1671–1675

Yang SC. Owen-Schaub L. Mendiguren-Rodriguez A. Grimm EA. Hong WK and Roth JA (1990) Combination immunotherapy for non small cell lung cancer. Results with interleukin-2 and tumor necrosis factor-alpha. *J Thoracic Cardiovasc Surg* **99**: 8–12 (discussion 12–13)

Yoshino I. Yano T. Murata M. Ishida T. Sugimachi K. Kimura G and Nomoto K (1992) Tumour-reactive T cells accumulate in lung cancer tissues but fail due to respond to tumour cell derived factor. *Cancer Res* **52**: 775–781

© Cancer Research Campaign 1998


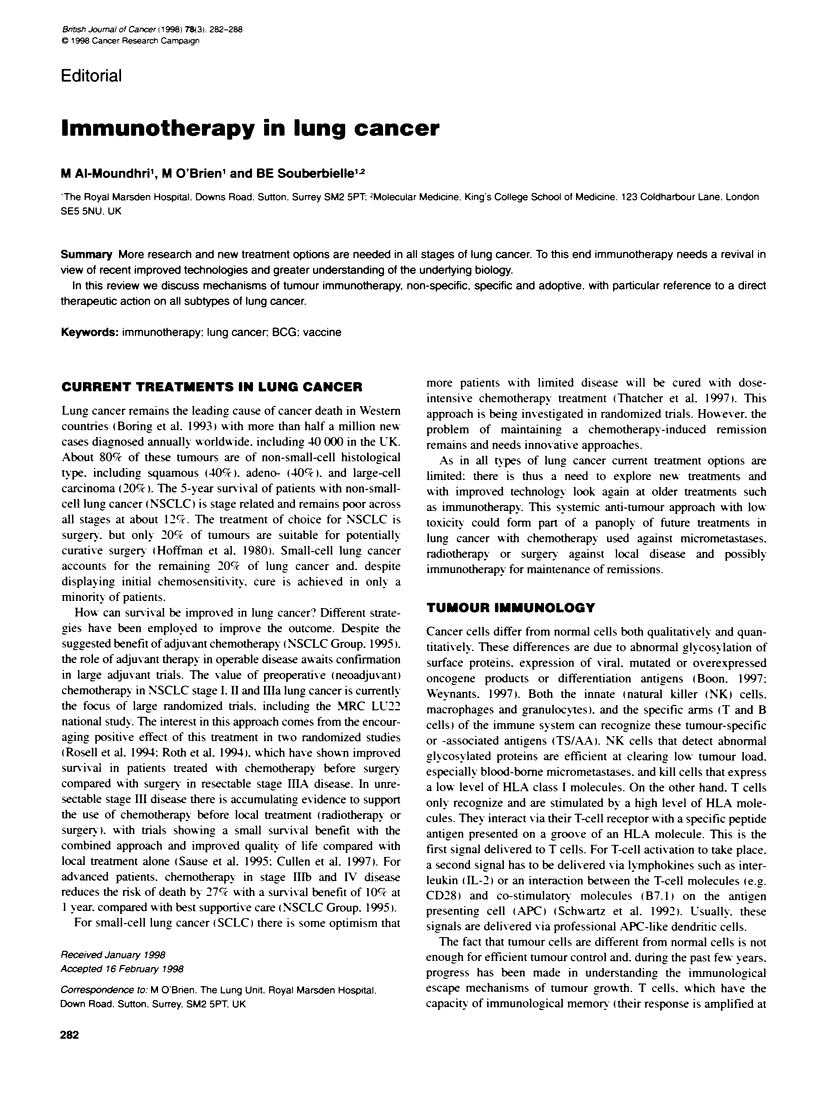

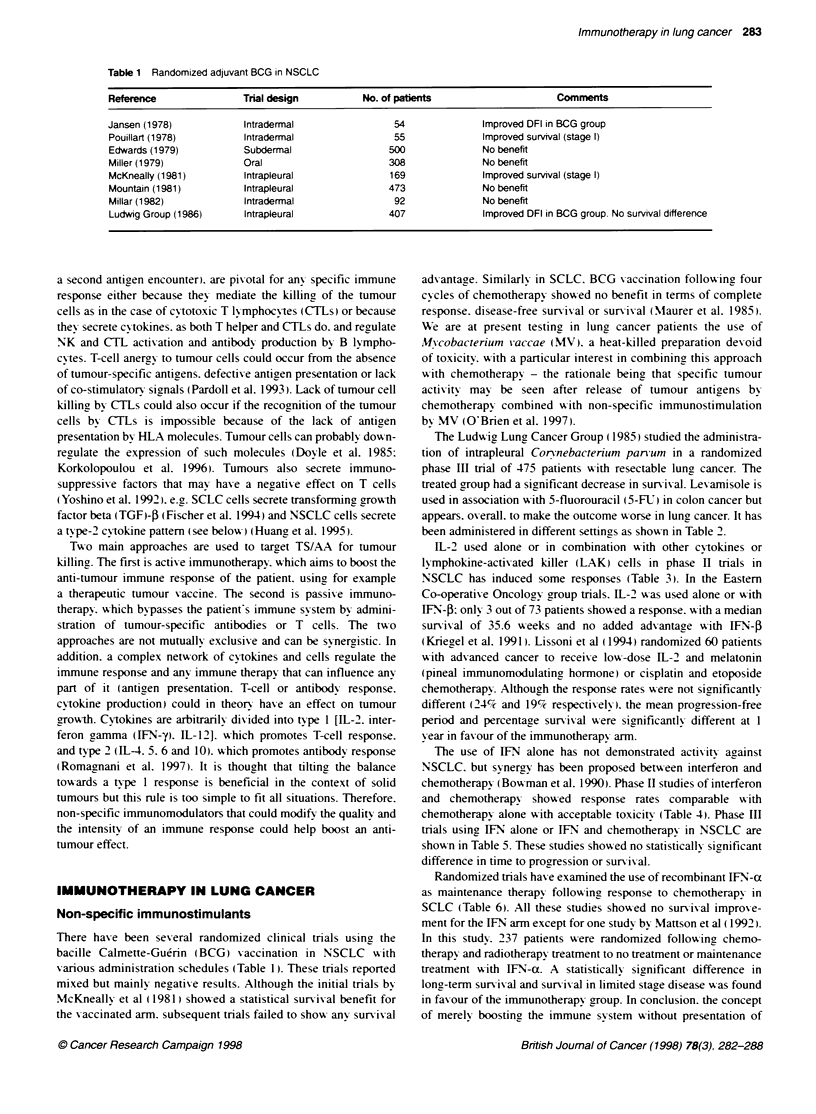

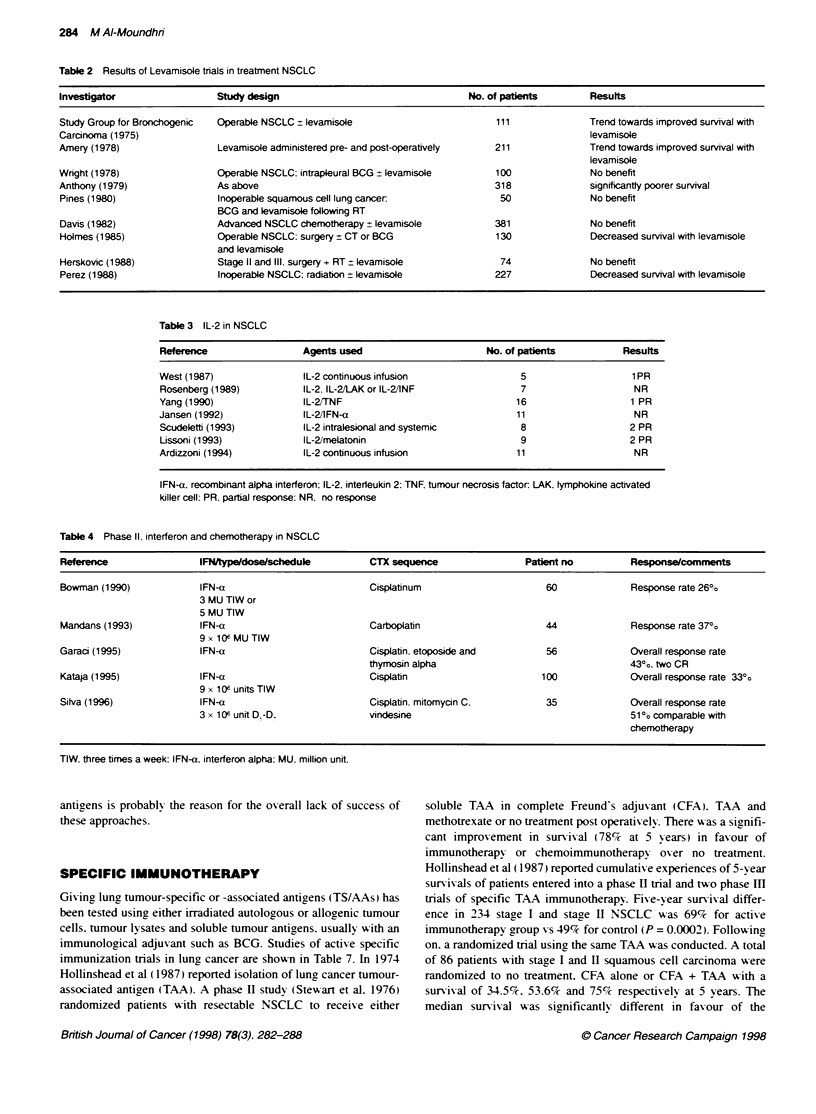

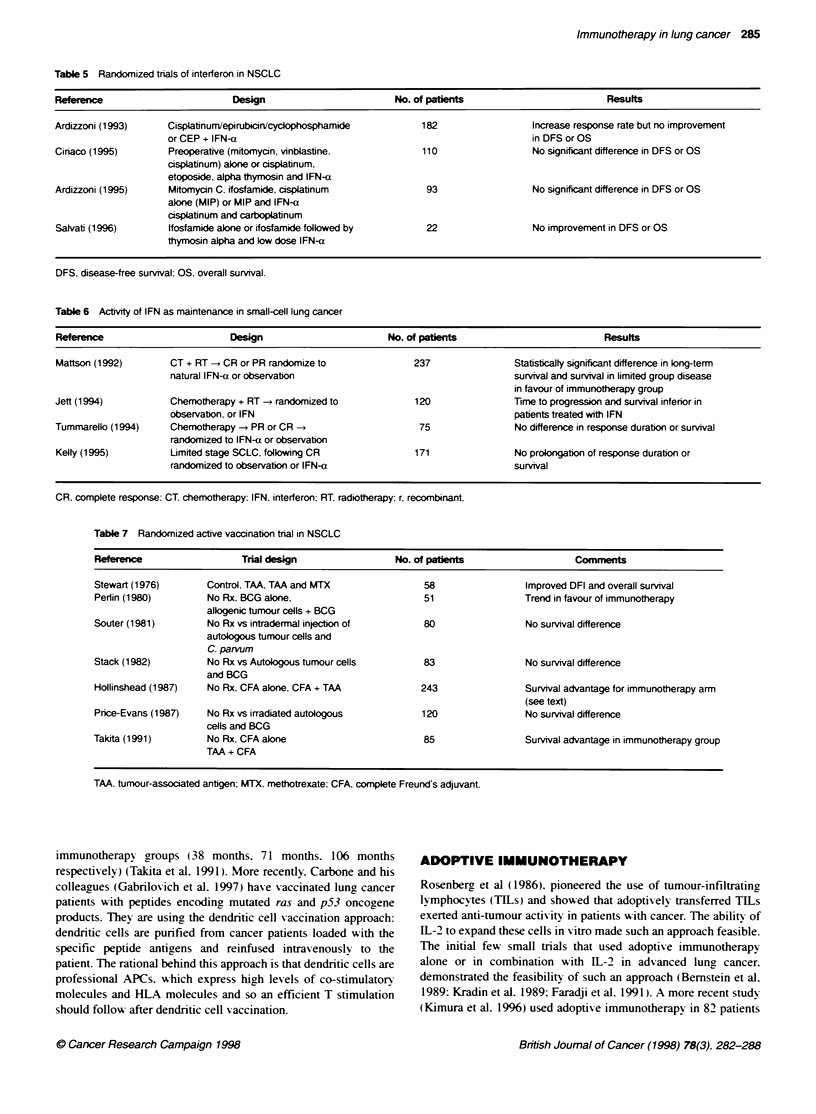

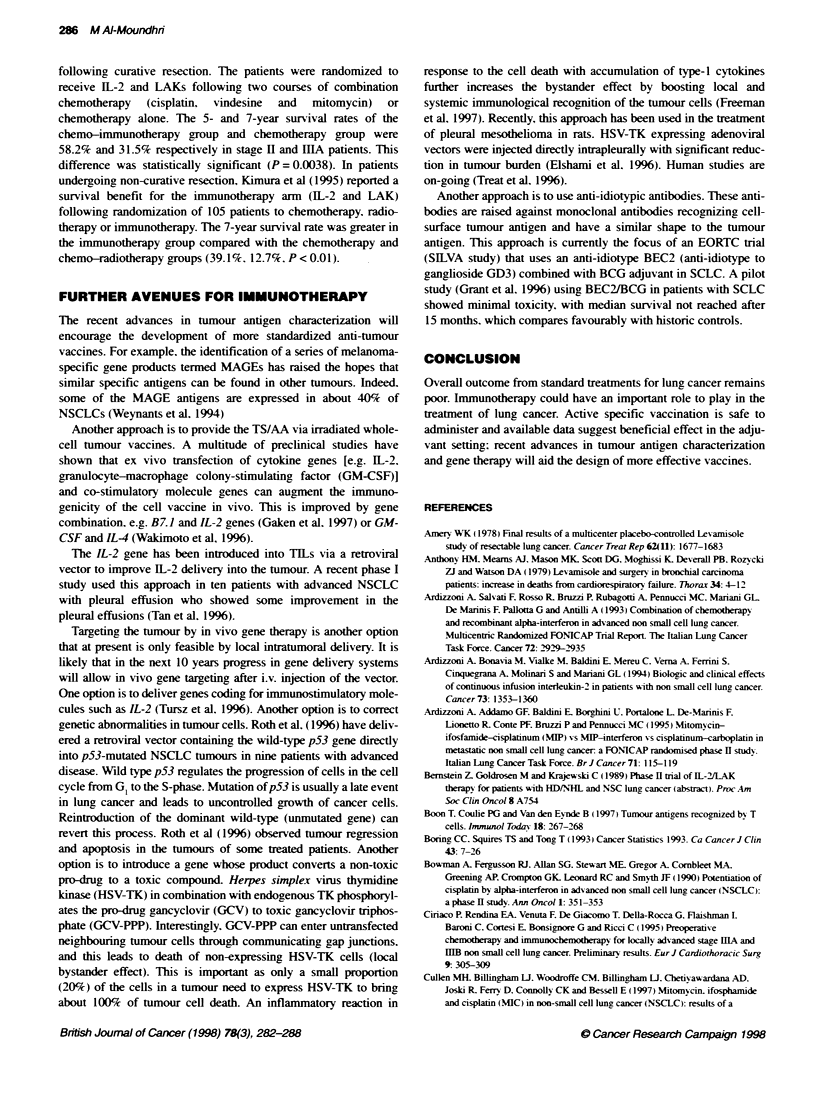

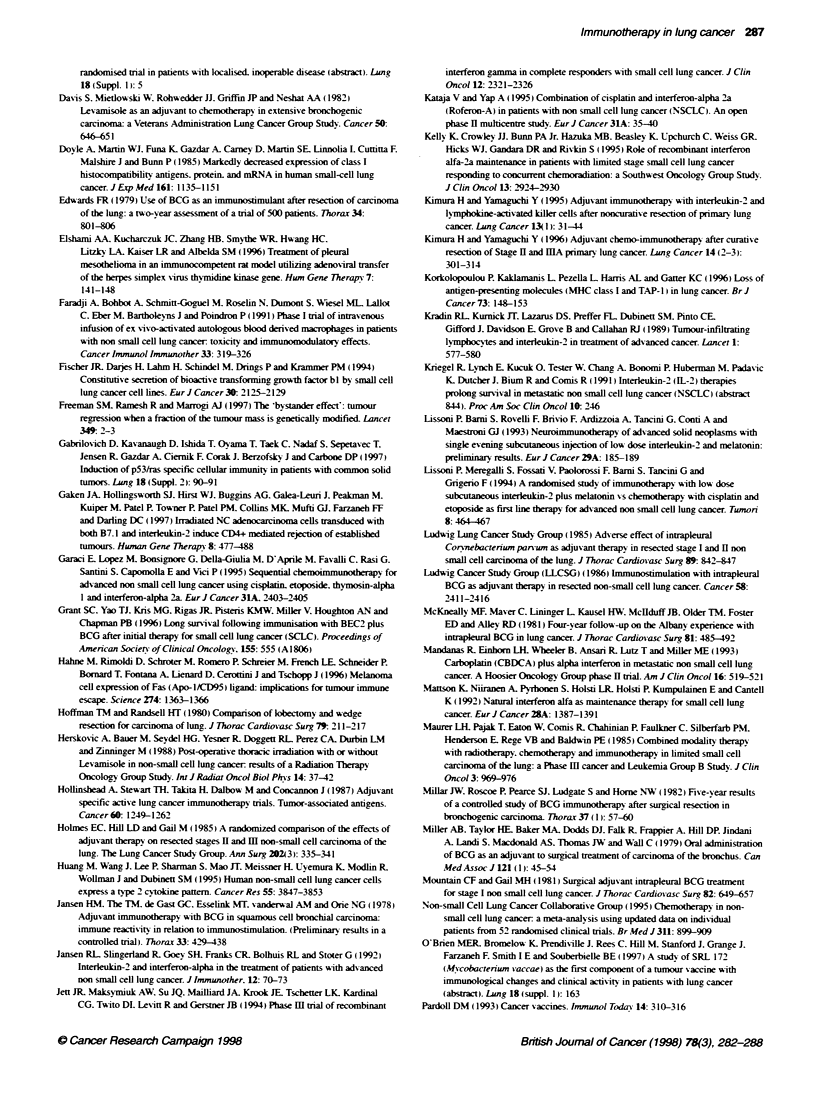

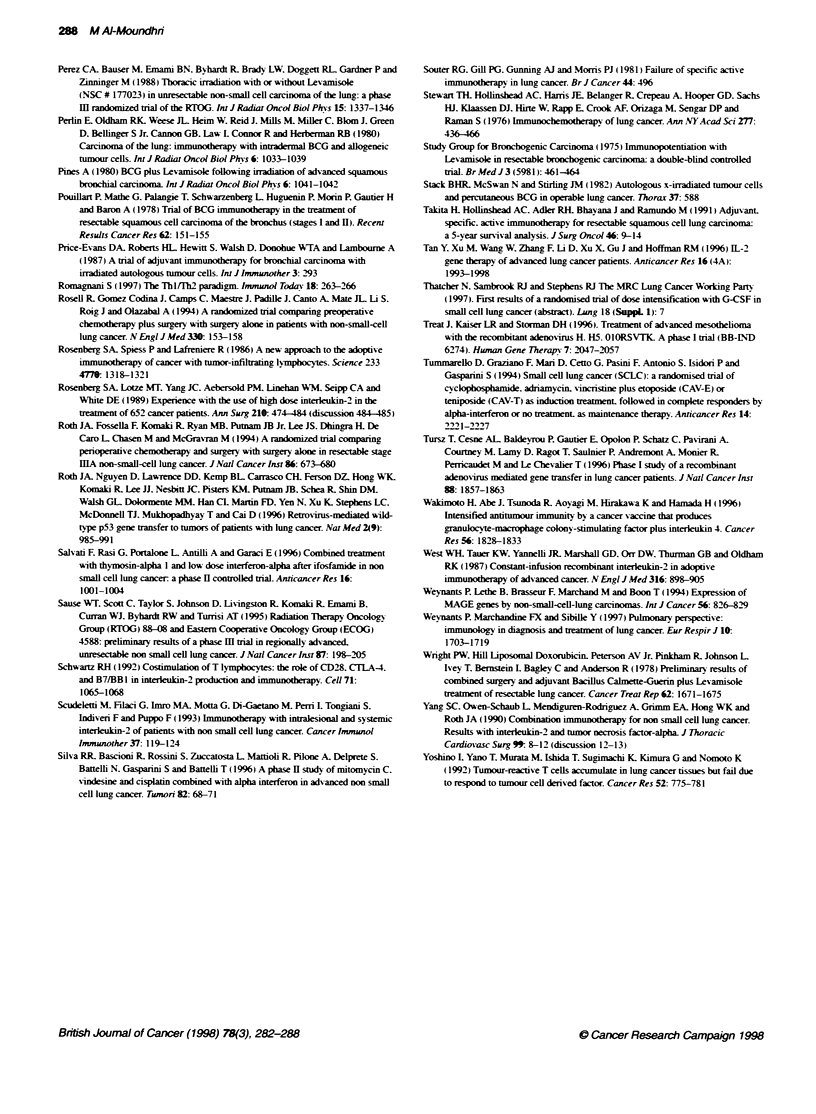


## References

[OCR_00561] Anthony H. M., Mearns A. J., Mason M. K., Scott D. G., Moghissi K., Deverall P. B., Rozycki Z. J., Watson D. A. (1979). Levamisole and surgery in bronchial carcinoma patients: increase in deaths from cardiorespiratory failure.. Thorax.

[OCR_00581] Ardizzoni A., Addamo G. F., Baldini E., Borghini U., Portalone L., De Marinis F., Lionetto R., Conte P. F., Bruzzi P., Pennucci M. C. (1995). Mitomycin-ifosfamide-cisplatinum (MIP) vs MIP-interferon vs cisplatinum-carboplatin in metastatic non-small-cell lung cancer: a FONICAP randomised phase II study. Italian Lung Cancer Task Force.. Br J Cancer.

[OCR_00571] Ardizzoni A., Bonavia M., Viale M., Baldini E., Mereu C., Verna A., Ferrini S., Cinquegrana A., Molinari S., Mariani G. L. (1994). Biologic and clinical effects of continuous infusion interleukin-2 in patients with non-small cell lung cancer.. Cancer.

[OCR_00563] Ardizzoni A., Salvati F., Rosso R., Bruzzi P., Rubagotti A., Pennucci M. C., Mariani G. L., De Marinis F., Pallotta G., Antilli A. (1993). Combination of chemotherapy and recombinant alpha-interferon in advanced non-small cell lung cancer. Multicentric Randomized FONICAP Trial Report. The Italian Lung Cancer Task Force.. Cancer.

[OCR_00590] Boon T., Coulie P. G., Van den Eynde B. (1997). Tumor antigens recognized by T cells.. Immunol Today.

[OCR_00594] Boring C. C., Squires T. S., Tong T. (1993). Cancer statistics, 1993.. CA Cancer J Clin.

[OCR_00601] Bowman A., Fergusson R. J., Allan S. G., Stewart M. E., Gregor A., Cornbleet M. A., Greening A. P., Crompton G. K., Leonard R. C., Smyth J. F. (1990). Potentiation of cisplatin by alpha-interferon in advanced non-small cell lung cancer (NSCLC): a phase II study.. Ann Oncol.

[OCR_00604] Ciriaco P., Rendina E. A., Venuta F., De Giacomo T., Della Rocca G., Flaishman I., Baroni C., Cortesi E., Bonsignore G., Ricci C. (1995). Preoperative chemotherapy and immunochemotherapy for locally advanced stage IIIA and IIIB non small cell lung cancer. Preliminary results.. Eur J Cardiothorac Surg.

[OCR_00625] Davis S., Mietlowski W., Rohwedder J. J., Griffin J. P., Neshat A. A. (1982). Levamisole as an adjuvant to chemotherapy in extensive bronchogenic carcinoma: a Veterans Administration Lung Cancer Group Study.. Cancer.

[OCR_00634] Doyle A., Martin W. J., Funa K., Gazdar A., Carney D., Martin S. E., Linnoila I., Cuttitta F., Mulshine J., Bunn P. (1985). Markedly decreased expression of class I histocompatibility antigens, protein, and mRNA in human small-cell lung cancer.. J Exp Med.

[OCR_00639] Edwards F. R. (1979). Use of BCG as an immunostimulant after resection of carcinoma of the lung: a two-year assessment of a trial of 500 patients.. Thorax.

[OCR_00644] Elshami A. A., Kucharczuk J. C., Zhang H. B., Smythe W. R., Hwang H. C., Litzky L. A., Kaiser L. R., Albelda S. M. (1996). Treatment of pleural mesothelioma in an immunocompetent rat model utilizing adenoviral transfer of the herpes simplex virus thymidine kinase gene.. Hum Gene Ther.

[OCR_00654] Faradji A., Bohbot A., Schmitt-Goguel M., Roeslin N., Dumont S., Wiesel M. L., Lallot C., Eber M., Bartholeyns J., Poindron P. (1991). Phase I trial of intravenous infusion of ex-vivo-activated autologous blood-derived macrophages in patients with non-small-cell lung cancer: toxicity and immunomodulatory effects.. Cancer Immunol Immunother.

[OCR_00662] Fischer J. R., Darjes H., Lahm H., Schindel M., Drings P., Krammer P. H. (1994). Constitutive secretion of bioactive transforming growth factor beta 1 by small cell lung cancer cell lines.. Eur J Cancer.

[OCR_00680] Gaken J. A., Hollingsworth S. J., Hirst W. J., Buggins A. G., Galea-Lauri J., Peakman M., Kuiper M., Patel P., Towner P., Patel P. M. (1997). Irradiated NC adenocarcinoma cells transduced with both B7.1 and interleukin-2 induce CD4+-mediated rejection of established tumors.. Hum Gene Ther.

[OCR_00686] Garaci E., Lopez M., Bonsignore G., Della Giulia M., D'Aprile M., Favalli C., Rasi G., Santini S., Capomolla E., Vici P. (1995). Sequential chemoimmunotherapy for advanced non-small cell lung cancer using cisplatin, etoposide, thymosin-alpha 1 and interferon-alpha 2a.. Eur J Cancer.

[OCR_00696] Hahne M., Rimoldi D., Schröter M., Romero P., Schreier M., French L. E., Schneider P., Bornand T., Fontana A., Lienard D. (1996). Melanoma cell expression of Fas(Apo-1/CD95) ligand: implications for tumor immune escape.. Science.

[OCR_00706] Herskovic A., Bauer M., Seydel H. G., Yesner R., Doggett R. L., Perez C. A., Durbin L. M., Zinninger M. (1988). Post-operative thoracic irradiation with or without levamisole in non-small cell lung cancer: results of a Radiation Therapy Oncology Group Study.. Int J Radiat Oncol Biol Phys.

[OCR_00702] Hoffmann T. H., Ransdell H. T. (1980). Comparison of lobectomy and wedge resection for carcinoma of the lung.. J Thorac Cardiovasc Surg.

[OCR_00714] Hollinshead A., Stewart T. H., Takita H., Dalbow M., Concannon J. (1987). Adjuvant specific active lung cancer immunotherapy trials. Tumor-associated antigens.. Cancer.

[OCR_00719] Holmes E. C., Hill L. D., Gail M. (1985). A randomized comparison of the effects of adjuvant therapy on resected stages II and III non-small cell carcinoma of the lung. The Lung Cancel Study Group.. Ann Surg.

[OCR_00725] Huang M., Wang J., Lee P., Sharma S., Mao J. T., Meissner H., Uyemura K., Modlin R., Wollman J., Dubinett S. M. (1995). Human non-small cell lung cancer cells express a type 2 cytokine pattern.. Cancer Res.

[OCR_00727] Jansen H. M., The T. H., de Gast G. C., Esselink M. T., van der Wal A. M., Orie N. G. (1978). Adjuvant immunotherapy with BCG in squamous-cell bronchial carcinoma. Immune-reactivity in relation to immunostimulation (preliminary results in a controlled trial).. Thorax.

[OCR_00734] Jansen R. L., Slingerland R., Goey S. H., Franks C. R., Bolhuis R. L., Stoter G. (1992). Interleukin-2 and interferon-alpha in the treatment of patients with advanced non-small-cell lung cancer.. J Immunother (1991).

[OCR_00739] Jett J. R., Maksymiuk A. W., Su J. Q., Mailliard J. A., Krook J. E., Tschetter L. K., Kardinal C. G., Twito D. I., Levitt R., Gerstner J. B. (1994). Phase III trial of recombinant interferon gamma in complete responders with small-cell lung cancer.. J Clin Oncol.

[OCR_00746] Kataja V., Yap A. (1995). Combination of cisplatin and interferon-alpha 2a (Roferon-A) in patients with non-small cell lung cancer (NSCLC). An open phase II multicentre study.. Eur J Cancer.

[OCR_00751] Kelly K., Crowley J. J., Bunn P. A., Hazuka M. B., Beasley K., Upchurch C., Weiss G. R., Hicks W. J., Gandara D. R., Rivkin S. (1995). Role of recombinant interferon alfa-2a maintenance in patients with limited-stage small-cell lung cancer responding to concurrent chemoradiation: a Southwest Oncology Group study.. J Clin Oncol.

[OCR_00764] Kimura H., Yamaguchi Y. (1996). Adjuvant chemo-immunotherapy after curative resection of Stage II and IIIA primary lung cancer.. Lung Cancer.

[OCR_00759] Kimura H., Yamaguchi Y. (1995). Adjuvant immunotherapy with interleukin 2 and lymphokine-activated killer cells after noncurative resection of primary lung cancer.. Lung Cancer.

[OCR_00771] Korkolopoulou P., Kaklamanis L., Pezzella F., Harris A. L., Gatter K. C. (1996). Loss of antigen-presenting molecules (MHC class I and TAP-1) in lung cancer.. Br J Cancer.

[OCR_00774] Kradin R. L., Kurnick J. T., Lazarus D. S., Preffer F. I., Dubinett S. M., Pinto C. E., Gifford J., Davidson E., Grove B., Callahan R. J. (1989). Tumour-infiltrating lymphocytes and interleukin-2 in treatment of advanced cancer.. Lancet.

[OCR_00789] Lissoni P., Barni S., Rovelli F., Brivio F., Ardizzoia A., Tancini G., Conti A., Maestroni G. J. (1993). Neuroimmunotherapy of advanced solid neoplasms with single evening subcutaneous injection of low-dose interleukin-2 and melatonin: preliminary results.. Eur J Cancer.

[OCR_00818] Mandanas R., Einhorn L. H., Wheeler B., Ansari R., Lutz T., Miller M. E. (1993). Carboplatin (CBDCA) plus alpha interferon in metastatic non-small cell lung cancer. A Hoosier Oncology Group phase II trial.. Am J Clin Oncol.

[OCR_00823] Mattson K., Niiranen A., Pyrhönen S., Holsti L. R., Holsti P., Kumpulainen E., Cantell K. (1992). Natural interferon alfa as maintenance therapy for small cell lung cancer.. Eur J Cancer.

[OCR_00829] Maurer L. H., Pajak T., Eaton W., Comis R., Chahinian P., Faulkner C., Silberfarb P. M., Henderson E., Rege V. B., Baldwin P. E. (1985). Combined modality therapy with radiotherapy, chemotherapy, and immunotherapy in limited small-cell carcinoma of the lung: a Phase III cancer and Leukemia Group B Study.. J Clin Oncol.

[OCR_00812] McKneally M. F., Maver C., Lininger L., Kausel H. W., McIlduff J. B., Older T. M., Foster E. D., Alley R. D. (1981). Four-year follow-up on the Albany experience with intrapleural BCG in lung cancer.. J Thorac Cardiovasc Surg.

[OCR_00839] Miller A. B., Taylor H. E., Baker M. A., Dodds D. J., Falk R., Frappier A., Hill D. P., Jindani A., Landi S., Macdonald A. S. (1979). Oral administration of BCG as an adjuvant to surgical treatment of carcinoma of the bronchus.. Can Med Assoc J.

[OCR_00861] Pardoll D. M. (1993). Cancer vaccines.. Immunol Today.

[OCR_00869] Perez C. A., Bauer M., Emami B. N., Byhardt R., Brady L. W., Doggett R. L., Gardner P., Zinninger M. (1988). Thoracic irradiation with or without levamisole (NSC #177023) in unresectable non-small cell carcinoma of the lung: a phase III randomized trial of the RTOG.. Int J Radiat Oncol Biol Phys.

[OCR_00873] Perlin E., Oldham R. K., Weese J. L., Heim W., Reid J., Mills M., Miller C., Blom J., Green D., Bellinger S. (1980). Carcinoma of the lung: immunotherapy with intradermal BCG and allogeneic tumor cells.. Int J Radiat Oncol Biol Phys.

[OCR_00880] Pines A. (1980). BCG plus levamisole following irradiation of advanced squamous bronchial carcinoma.. Int J Radiat Oncol Biol Phys.

[OCR_00896] Romagnani S. (1997). The Th1/Th2 paradigm.. Immunol Today.

[OCR_00900] Rosell R., Gómez-Codina J., Camps C., Maestre J., Padille J., Cantó A., Mate J. L., Li S., Roig J., Olazábal A. (1994). A randomized trial comparing preoperative chemotherapy plus surgery with surgery alone in patients with non-small-cell lung cancer.. N Engl J Med.

[OCR_00910] Rosenberg S. A., Lotze M. T., Yang J. C., Aebersold P. M., Linehan W. M., Seipp C. A., White D. E. (1989). Experience with the use of high-dose interleukin-2 in the treatment of 652 cancer patients.. Ann Surg.

[OCR_00907] Rosenberg S. A., Spiess P., Lafreniere R. (1986). A new approach to the adoptive immunotherapy of cancer with tumor-infiltrating lymphocytes.. Science.

[OCR_00922] Roth J. A., Nguyen D., Lawrence D. D., Kemp B. L., Carrasco C. H., Ferson D. Z., Hong W. K., Komaki R., Lee J. J., Nesbitt J. C. (1996). Retrovirus-mediated wild-type p53 gene transfer to tumors of patients with lung cancer.. Nat Med.

[OCR_00932] Salvati F., Rasi G., Portalone L., Antilli A., Garaci E. (1996). Combined treatment with thymosin-alpha1 and low-dose interferon-alpha after ifosfamide in non-small cell lung cancer: a phase-II controlled trial.. Anticancer Res.

[OCR_00938] Sause W. T., Scott C., Taylor S., Johnson D., Livingston R., Komaki R., Emami B., Curran W. J., Byhardt R. W., Turrisi A. T. (1995). Radiation Therapy Oncology Group (RTOG) 88-08 and Eastern Cooperative Oncology Group (ECOG) 4588: preliminary results of a phase III trial in regionally advanced, unresectable non-small-cell lung cancer.. J Natl Cancer Inst.

[OCR_00946] Schwartz R. H. (1992). Costimulation of T lymphocytes: the role of CD28, CTLA-4, and B7/BB1 in interleukin-2 production and immunotherapy.. Cell.

[OCR_00953] Scudeletti M., Filaci G., Imro M. A., Motta G., Di Gaetano M., Pierri I., Tongiani S., Indiveri F., Puppo F. (1993). Immunotherapy with intralesional and systemic interleukin-2 of patients with non-small-cell lung cancer.. Cancer Immunol Immunother.

[OCR_00957] Silva R. R., Bascioni R., Rossini S., Zuccatosta L., Mattioli R., Pilone A., Delprete S., Battelli N., Gasparini S., Battelli T. (1996). A phase II study of mitomycin C, vindesine and cisplatin combined with alpha interferon in advanced non-small cell lung cancer.. Tumori.

[OCR_00963] Souter R. G., Gill P. G., Gunning A. J., Morris P. J. (1981). Failure of specific active immunotherapy in lung cancer.. Br J Cancer.

[OCR_00979] Stack B. H., McSwan N., Stirling J. M., Hole D. J., Spilg W. G., McHattie I., Elliott J. A., Gillis C. R., Turner M. A., White R. G. (1982). Autologous x-irradiated tumour cells and percutaneous BCG in operable lung cancer.. Thorax.

[OCR_00971] Stewart T. H., Hollinshead A. C., Harris J. E., Belanger R., Crepeau A., Hooper G. D., Sachs H. J., Klaassen D. J., Hirte W., Rapp E. (1976). Immunochemotherapy of lung cancer.. Ann N Y Acad Sci.

[OCR_00845] (1981). Surgical adjuvant intrapleural BCG treatment for stage I non-small cell lung cancer. Preliminary report of the National Cancer Institute Lung Cancer Study Group.. J Thorac Cardiovasc Surg.

[OCR_00985] Takita H., Hollinshead A. C., Adler R. H., Bhayana J., Ramundo M., Moskowitz R., Rao U. N., Raman S. (1991). Adjuvant, specific, active immunotherapy for resectable squamous cell lung carcinoma: a 5-year survival analysis.. J Surg Oncol.

[OCR_00990] Tan Y., Xu M., Wang W., Zhang F., Li D., Xu X., Gu J., Hoffman R. M. (1996). IL-2 gene therapy of advanced lung cancer patients.. Anticancer Res.

[OCR_00998] Treat J., Kaiser L. R., Sterman D. H., Litzky L., Davis A., Wilson J. M., Albelda S. M. (1996). Treatment of advanced mesothelioma with the recombinant adenovirus H5.010RSVTK: a phase 1 trial (BB-IND 6274).. Hum Gene Ther.

[OCR_01003] Tummarello D., Graziano F., Mari D., Cetto G., Pasini F., Antonio S., Isidori P., Gasparini S. (1994). Small cell lung cancer (SCLC): a randomized trial of cyclophosphamide, adriamycin, vincristine plus etoposide (CAV-E) or teniposide (CAV-T) as induction treatment, followed in complete responders by alpha-interferon or no treatment, as maintenance therapy.. Anticancer Res.

[OCR_01016] Tursz T., Cesne A. L., Baldeyrou P., Gautier E., Opolon P., Schatz C., Pavirani A., Courtney M., Lamy D., Ragot T. (1996). Phase I study of a recombinant adenovirus-mediated gene transfer in lung cancer patients.. J Natl Cancer Inst.

[OCR_01023] Wakimoto H., Abe J., Tsunoda R., Aoyagi M., Hirakawa K., Hamada H. (1996). Intensified antitumor immunity by a cancer vaccine that produces granulocyte-macrophage colony-stimulating factor plus interleukin 4.. Cancer Res.

[OCR_01031] West W. H., Tauer K. W., Yannelli J. R., Marshall G. D., Orr D. W., Thurman G. B., Oldham R. K. (1987). Constant-infusion recombinant interleukin-2 in adoptive immunotherapy of advanced cancer.. N Engl J Med.

[OCR_01033] Weynants P., Lethé B., Brasseur F., Marchand M., Boon T. (1994). Expression of mage genes by non-small-cell lung carcinomas.. Int J Cancer.

[OCR_01038] Weynants P., Marchandise F. X., Sibille Y. (1997). Pulmonary perspective: immunology in diagnosis and treatment of lung cancer.. Eur Respir J.

[OCR_01044] Wright P. W., Hill L. D., Peterson A. V., Pinkham R., Johnson L., Ivey T., Bernstein I., Bagley C., Anderson R. (1978). Preliminary results of combined surgery and adjuvant Bacillus Calmette-Guérin plus levamisole treatment of resectable lung cancer.. Cancer Treat Rep.

[OCR_01047] Yang S. C., Owen-Schaub L., Mendiguren-Rodriguez A., Grimm E. A., Hong W. K., Roth J. A. (1990). Combination immunotherapy for non-small cell lung cancer. Results with interleukin-2 and tumor necrosis factor-alpha.. J Thorac Cardiovasc Surg.

[OCR_00777] Yemini M., Borenstein R., Dreazen E., Apelman Z., Mogilner B. M., Kessler I., Lancet M. (1985). Prevention of premature labor by 17 alpha-hydroxyprogesterone caproate.. Am J Obstet Gynecol.

[OCR_01053] Yoshino I., Yano T., Murata M., Ishida T., Sugimachi K., Kimura G., Nomoto K. (1992). Tumor-reactive T-cells accumulate in lung cancer tissues but fail to respond due to tumor cell-derived factor.. Cancer Res.

